# Supplementary material for: General treatment for stereo-dynamics of state-to-state chemi-ionization reactions
Source: Commun Chem. 2020 May 25;3:64. doi: 10.1038/s42004-020-0312-3 (PMC9814700; doi:10.1038/s42004-020-0312-3)
Supplement: Supplementary file 1 — Supplementary Information [file 42004_2020_312_MOESM1_ESM.pdf]

SUPPLEMENTARY INFORMATION

General treatment for stereo-dynamics of state-to-state chemi-ionization reactions

Stefano Falcinelli,[1] Franco Vecchiocattivi[1] and Fernando Pirani,[2]*

[1]Department of Civil and Environmental Engineering,

University of Perugia, Via G. Duranti 93, 06125 Perugia, Italy.

[2]Department of Chemistry, Biology and Biotechnologies, University of Perugia,

Via Elce di Sotto 8, 06123 Perugia, Italy.

Supplementary Methods

Additional details on the potential formulation

The adopted formulation of the real component V_t of the optical potential, providing for the entrance channels the dependence of the isotropic interaction on the reagent separation distance R, leads to this expression[1]:

$$V_t(R) = S(R)\, V(R)^{neut.-neut.} + \big(1 - S(R)\big)\, V(R)^{ion-neut.} \tag{1}$$

where $V(R)^{neut.-neut.}$ and $V(R)^{ion-neut.}$ are represented by the Improved Lennard Jones (ILJ) function, whose general form is[2]:

$$V_{ILJ}(R) = \varepsilon\left[\frac{m}{n(R)-m}\left(\frac{R_m}{R}\right)^{n(R)} - \frac{n(R)}{n(R)-m}\left(\frac{R_m}{R}\right)^{m}\right] \tag{2}$$

with

$$n(R) = \beta + 4\left(\frac{R}{R_m}\right)^2 \quad (3)$$

Here ε is the potential well depth and R_m is its location, while $n(R)$ defines the hardness of the repulsive wall and the radial modulation of the attraction. The switching function $S(R)$, that accounts for the transition from the neutral-neutral to the ion-neutral representation of the interaction (see left upper panel of Supplementary Fig. 1), as previously[1] it has been defined as

$$S(R) = \frac{1}{1+ e^{\left(\frac{R_O - R}{d}\right)}} \quad (4)$$

Here R_o is the distance where the two combined limiting potential forms have the same weight, while d describes how fast the transition occurs.

The R dependence of the isotropic component of the interaction in the exit channel is represented again by an ILJ function. Adopted parameter values are reported in Supplementary Table 1. However, for the complete representation of V_t it is necessary to take into account all aspects discussed in the main text.

Additional Details on the Ne^*-Kr phenomenology

The experiments performed with the molecular beam (MB) technique provided total and partial ionization cross sections (σ)[3-7], associative to Penning (σ_{as}/σ_{pe}) ratios[8-10] and Penning Ionization Electron Spectra (PIES)[7,12-14]: crucially, all data have been measured under single collision condition and as a function of the collision energy.

It has to be noted that the theoretical treatment, presented in this paper and stimulated by the PIES measurements in our laboratory, is able to rationalize in a unifying picture all the observables quoted above, probing different features of the optical potential (see eq. (3) of the main text). In particular, as demonstrated below, the adopted methodology is able to reproduce,

in a state to state condition, all experimental data available for the Ne^*-Kr system obtained from our and other laboratories, including total and partial ionization cross sections and branching ratios between selected channels. Therefore, the proposed theoretical approach, which also includes within the same framework exchange and radiative mechanisms proposed in the past[15], is general and can be used to describe in a state to state condition the reactivity of all chemi-ionization reactions, including those involving molecules.

A schematic view of the MB apparatus available in our laboratory is given in the left upper panel of Supplementary Fig. 1.

PIES data and extracted peak area ratios reported in the same Figure confirm that the reaction probability is dependent on the reaction channel, identified by the spin-orbit state of neutral reactants and ionic products, defined by the total electronic quantum number J, and it is modulated by the collision energy. An overview of the global observed phenomenology, including PIESs and many other observables, suggests the existence of important selectivity in the reaction dynamics which is properly rationalized by the new method discussed in the main text.

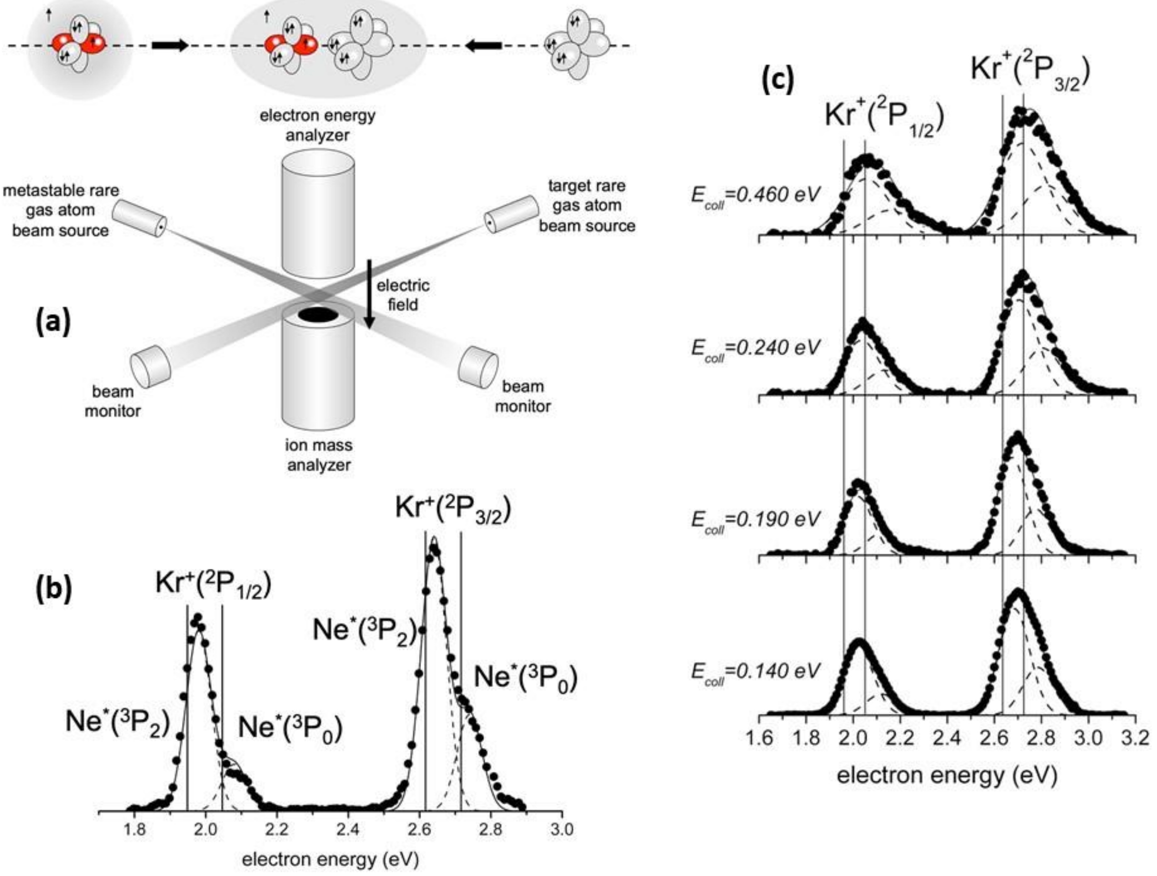

Supplementary Fig. 1

(a) A schematic view of the apparatus, where the primary beam of $Ne^*(^3P_J$, with J=2,0) atoms, emerging from an electron bombardment supersonic seeded source, crosses at right angles the secondary beam of Kr atoms. PIESs have been measured exploiting a hemispherical electron energy analyzer, while total, partial cross sections and branching ratios have been determined by mass spectrometry. **(b)** A Ne^*-Kr PIES measured at low collision energy (50 meV), where the contributions of four reaction channels, associated to two different spin-orbit states J of Ne^* neutral reactant (J_i = 2,0) and of $Kr^+(^2P_J)$ ionic product (J_f = 3/2, 1/2), have been resolved. Vertical continuous lines represent the peak positions, predicted for the ionization of Kr by Ne(I) photons, and the shift of observed maxima in measured PIES relates to structure and stability of the reaction transition state. The peak area ratios, defining the relative reaction yields of the four channels, have been also evaluated through the analysis performed adopting four independent Gaussian functions with the same width[14]. **(c)** PIESs measured as a function of the collision energy (vertical lines as in the left lower panel). Their analysis (see caption of left lower panel) emphasizes the dependence of the peak position and of the peak area ratios on the collision energy. In the Figure the depicted p-orbitals do not indicate a specific and mutual orientation but only the most probable orientation producing ionization.

Note that in the right panel of Supplementary Fig. 1 is shown a comparison between state to state cross section ratios, predicted by the treatment discussed in the main text and referred to specific spin orbit sublevels of reactants and products, with peak area ratios extracted from PIESs measured as a function of the collision energy[14,16]. For two of the four ratios such a comparison appears to be only in semi-quantitative agreement. However, considering the difficulties involved in the separation of individual spin orbit contributions from measured energy dependence of PIESs, also for such quantities the theoretical description appears to be consistent with the experimental observables.

The next Supplementary Fig. 2 shows the manifold of the potential energy curves associated to V_t, describing all adiabatic states for both entrance and exit channels. The same Figure, depicting half-filled and filled orbitals of reagents and products, justifies the origin of the configuration interaction by charge transfer which plays a crucial role in the control of the reaction dynamics.

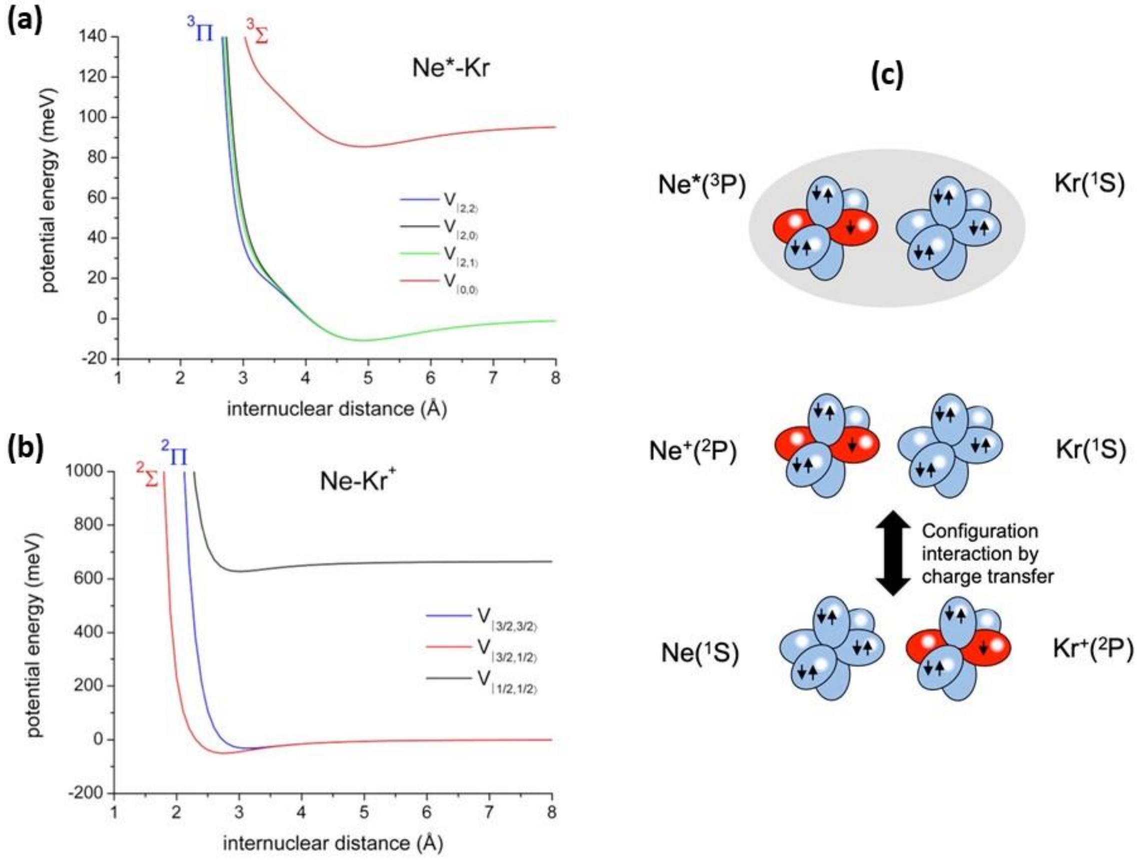

Supplementary Fig. 2

The real part of W represented by *adiabatic* potential energy curves that are formulated in an internally consistent way for both entrance **(a)** and exit **(b)** channels, as described in detail in the main text and whose potential parameters are given in Supplementary Table 1 below. The interaction anisotropy at long range depends on the polarizability anisotropy of Ne^* atom, while, at intermediate and short range, it arises from anisotropic charge transfer (CT) contributions associated to configuration interaction between states differing for one electron exchange. **(c)** Representation of the configuration interaction, between states of entrance and exit channels differing for one electron exchange, which defines the CT contribution for Σ symmetry states. The corresponding component for Π symmetry states is much smaller (~1/5)[16] because of the reduced overlap integral between atomic half-filled orbital exchanging the electron and aligned orthogonal to R. In the Figure the depicted p-orbitals do not indicate a specific and mutual orientation but only the most probable orientation producing ionization.

Fundamental aspects of the potential formulation are discussed in the main text and additional details presented in the previous section of present SI, while the complete list of parameters is given in Supplementary Table 1. The values of the parameters have been obtained from a

semi-empirical method founded on the ample phenomenology of the interactions of open-shell “P” atoms (particularly halogen atoms). They have been investigated in detail with scattering experiments, performed with state selected atomic beams and analyzed with a proper theoretical treatment (see for instance refs. 17-18 and references therein). The Supplementary Fig. 2 depicts also some *adiabatic* electronic rearrangements and the configuration interaction between molecular states of the same symmetry coupled by CT components, which represent the main contributions to the interaction anisotropy in the *adiabatic* framework.

Supplementary Table 1

The potential parameters of the real part V_t of the optical potential for entrance and exit channels of Ne^*-Kr system, used to calculate the adiabatic potential energy curves reported in Supplementary Fig. 2.

		Ne^*-Kr	Ne^+-Kr	Ne-Kr^+
Isotropic component	ε *(meV)*	9.0	209.0	37.5
	R_m*(Å)*	5.18	2.86	3.00
	β	6.7	8.0	8.5
	R_0*(Å)*	3.85		
	d (Å)	0.55		
Anisotropic component	*A (meV)*		1.6741×10^7	
	α *(Å^{-1})*		4.32	
	C_2 *(meV×Å^6)*			1850.

Supplementary References

[1] B. G. Brunetti, P. Candori, S. Falcinelli, F. Pirani, F. Vecchiocattivi, *J. Chem. Phys.* **2013,** *139***,** 164305.

[2] F. Pirani, S. Brizi, L. F. Ron*caratti, P. Casavecchia, D. Cappelletti, F. Vecchiocattivi, Phys. Chem. Chem. Phys.* **2008**, *10*, 5489-5503.

[3] H. Hotop, A. Niehaus, *Chem. Phys. Lett.* **1971**, *8*, 497-500.

[4] V. Hoffmann, H. Morgner, *J. Phys. B: Atom. Molec. Phys.* **1979**, *12*, 2857-2874.

[5] K. Ohno, H. Mutoh, Y. Harada, *J. Am. Chem. Soc.* **1983**, *105*, 4555-4561.

[6] K. Ohno, *Bull. Chem. Soc. Japan* **2004**, 77, 887-908.

[7] B. G. Brunetti, P. Candori, D. Cappelletti, S. Falcinelli, F. Pirani, D. Stranges, F. Vecchiocattivi, *Chem. Phys. Lett.* **2012**, *539-540*, 19-23.

[8] S. D. S. Gordon, J. Zou, S. Tanteri, J. Jankunas, A. Osterwalder, *Phys. Rev. Lett.* **2017**, *119***,** 053001.

[9] S. D. S. Gordon, J. J. Omiste, J. Zou, S. Tanteri, P. Brumer, A. Osterwalder, *Nat. Chem.* **2018**, *10*, 1190-1195.

[10] J. Zou, S. D. S. Gordon, S. Tanteri, A. Osterwalder, *J. Chem. Phys.* **2018**, *148*, 164310.

[11] S. D. S. Gordon and A. Osterwalder, *Phys. Chem. Chem. Phys.* **2019**, *21*, 14306-14310.

[12] H. Hotop, *J. Electron. Spectrosc. Relat. Phenom.* **1981**, *23*, 347-365.

[13] B. A. Jacobs, W. A. Rice, P. E. Siska, *J. Chem. Phys*. **2003**, *118***,** 3124-3130.

[14] B. Brunetti, Candori, S. Falcinelli, B. Lescop, G. Liuti, F. Pirani, F. Vecchiocattivi, *Eur. Phys. J. D* **2006**, *38***,** 21-27.

[15] W. H. Miller and H. Morgner, H., *J. Chem. Phys.* **1977**, *67*, 4923-4930.

[16] S. Falcinelli, F. Vecchiocattivi, F. Pirani, *Phys. Rev. Lett.* **2018**, *121*, 163403.

[17] E. E. Nikitin and R. N. Zare, *Mol. Phys.* **1994**, 82, 85-100.

[18] F. Pirani, G. S. Maciel, D. Cappelletti, V. Aquilanti, *Int. Rev. Phys. Chem.* **2006**, *25*, 165-199.
